# The Effects of Incidentally Learned Temporal and Spatial Predictability on Response Times and Visual Fixations during Target Detection and Discrimination

**DOI:** 10.1371/journal.pone.0094539

**Published:** 2014-04-14

**Authors:** Melissa R. Beck, S. Lee Hong, Amanda E. van Lamsweerde, Justin M. Ericson

**Affiliations:** 1 Louisiana State University, Baton Rouge, Louisiana, United States of America; 2 Ohio University, Athens, Ohio, United States of America; Centre de Neuroscience Cognitive, France

## Abstract

Responses are quicker to predictable stimuli than if the time and place of appearance is uncertain. Studies that manipulate target predictability often involve overt cues to speed up response times. However, less is known about whether individuals will exhibit faster response times when target predictability is embedded within the inter-trial relationships. The current research examined the combined effects of spatial and temporal target predictability on reaction time (RT) and allocation of overt attention in a sustained attention task. Participants responded as quickly as possible to stimuli while their RT and eye movements were measured. Target temporal and spatial predictability were manipulated by altering the number of: 1) different time intervals between a response and the next target; and 2) possible spatial locations of the target. The effects of target predictability on target detection (Experiment 1) and target discrimination (Experiment 2) were tested. For both experiments, shorter RTs as target predictability increased across both space and time were found. In addition, the influences of spatial and temporal target predictability on RT and the overt allocation of attention were task dependent; suggesting that effective orienting of attention relies on both spatial and temporal predictability. These results indicate that stimulus predictability can be increased without overt cues and detected purely through inter-trial relationships over the course of repeated stimulus presentations.

## Introduction

Attention can be more efficiently oriented if it is known when and where potential targets will appear (e.g., [1] [2]). Therefore, the level of uncertainty associated with the target of attention is an important factor for responding to the target quickly. It has long been proposed that the “channel capacity,” which is the amount of information that can be processed in a set period of time (often measured in bits/second), is constant for any given task (see [3]). Consequently, the more unpredictable the stimulus, the more information that needs to be processed prior to the response and thus, the slower the reaction times (RT). This general effect has been seen in both RT (cf. [4] [5]) and movement time (cf.[6]). Overt and explicit cues that increase the predictability of when and where a target will appear decrease the amount of information to be processed and RT. However, what is less understood is the extent to which temporal and spatial predictability can be incidentally learned through inter-trial relationships.

Increasing the spatial predictability of a target decreases RT to the onset of the target ([7] [8] [9]); that is, if it is known where a target is likely to appear, responses to the target are quicker. Posner [9] used a cueing task to demonstrate that spatial predictability affects attention. Specifically, when responding to the onset of a target, RTs are shorter when a valid cue (cue in the same location as the target) appears in one of the possible target locations before the onset of a target compared to when the cue is invalid (cue in a location that is different from the target). This decrease in RT for valid trials is known as the spatial cueing effect. Apart from exogenous cues (i.e. explicit cues at a targets location), endogenous cues, which must be interpreted by the observer (e.g. an arrow pointing to the target location), can also reduce RT. For example, participants are able to detect a moving object more quickly when the object follows an expected spatial trajectory behind an occluder in comparison to conditions where there is no expectation of the object's trajectory [2].

Temporal predictability has also been found to influence RT. Specifically, RTs for target detection are shorter if it is known when the target will appear [1] [10] [11] [12]. For example, Correa, Lupiáñez, Milliken, and Tudela [13] found that prompting participants with a temporal cue, which indicated whether the target onset would be early or late, resulted in shorter RTs during a target detection task. This benefit is not gained by cueing alone, as mismatched cues (i.e., an early cue when the target arrived late) led to slower RTs.

There is still debate about whether spatial and temporal effects are independent or if they interact with one another in target detection (press one key for any target) and target discrimination (press one of two keys depending on the identity of the target) tasks. Some argue for the independence of spatial and temporal attention, pointing out differences in brain activation during target detection tasks [10] [14]. Meanwhile, others have argued for their interdependence by highlighting similarities in brain activation for spatial and temporal attention [1] during target detection tasks. Girardi et al. [15] demonstrated that spatial cueing effects in both target detection and discrimination tasks depend on temporal predictability. The spatial cueing effect for an endogenous spatial cue (a centrally presented arrow) was larger when the stimulus onset asynchrony (SOA) between the target and the cue was probable. However, without a probable SOA, the cueing effect was not reliable. MacKay and Juola [16] reported only additive effects of spatial and temporal cues in a target discrimination task. However, Milliken, Lupianez, Roberts, and Stevanovski [17] found larger spatial cueing effects in a target discrimination task when temporal cues were present, but found no such relationship in their target detection task.

The current study investigated whether the probability of when and where stimuli are likely to appear can be incidentally learned based on the locations and ISIs (inter-stimulus interval) of previous targets. Spatial predictability has typically been manipulated using cueing paradigms where the cue is a non-target visual stimulus preceding the target [18]. However, spatial predictability can be incidentally learned based on associations between cue identity and the location of the target [19]. Temporal predictability can also be incidentally learned based on the pattern of SOAs on previous trials [19] [17]. There is evidence that human participants have the capacity to detect the probability of event occurrences that accumulate over repeated presentations [20] [21] [22] [23] [24] [25] [26]. However, to the authors' knowledge, no study to date has examined the ability to incidentally learn and use probability information in regards to both spatial and temporal information within the same continuous performance task.

The current study examined the relationship between temporal and spatial predictability in a modified version of the continuous performance task [27]. In Experiment 1, participants performed a detection task in which they pressed one key when the target (a red square) appeared on the screen. In Experiment 2, participants completed a discrimination task in which they pressed one of two keys depending on the identity of target (red square or green square). Temporal and spatial predictability of the target was varied from block-to-block by altering the number of: 1) different time intervals between a response and the next target (ISI), and 2) possible spatial locations of the target. In addition to measuring RT to the targets, orienting attention to the targets was also measured. Therefore, patterns of visual fixations during the task were measured in order to determine the: A) allocation of overt attention during the ISI and B) when the target was fixated relative to target onset. Data from these dependent variables revealed that spatial and temporal probability interact to orient attention allowing for faster responses in the target detection task, but have independent effects in the target discrimination task.

## Experiment 1

Experiment 1 tested the effects of spatial and temporal predictability on visual fixations and RTs during a target detection task. In this task participants were simply asked to respond with a button press as soon as they saw the target on screen.

To manipulate temporal predictability, the consistency of the latency between the offset of one target and the onset of the next target was varied. That is, the number of different ISIs was varied across blocks of trials within the detection task. In the high predictability temporal block, each target always appeared 1250 ms after the previous response. This was the only ISI that occurred throughout the block. In the medium and low predictability conditions, the number of different ISIs was increased to two and four, respectively. It is important to note, however, that the pattern of RTs across increasing ISIs has been shown to form a hazard function where RTs decrease as ISI increases [28] [29]. To test whether a potential confound resembling a hazard function existed, we examined RT differences between the ISI used in the high temporal predictability condition and those used in the low and medium temporal predictability conditions.

Spatial predictability was manipulated through the consistency of the spatial location of the target from one trial to the next. In the high predictability spatial condition, the target always appeared in the same location. In the medium and low conditions, the targets appeared in two or four possible locations, respectively. Participants were free to move their eyes throughout the experiment; thus, depending on the location of the eyes, any given target may fall within the fovea or the periphery. As spatial predictability decreases, there is a greater likelihood that target onset will occur in the periphery of a participant's visual field, leading to slower target detection [30] [31] [32] [33]. It is important to note the target was large and salient enough that it did not require direct foveation to be detected regardless of its location. As a result, one could effectively use a covert scanning strategy by holding visual fixation in the center of the screen to perform the target detection task effectively.

RT was measured by recording the time between when the target appeared until a response was given. It was predicted that participants would be able to learn and use the probability cues to allocate attention more effectively, which would lead to shorter RTs when spatial or temporal predictability were high. Furthermore, if there is interdependence between the two, the RTs should be particularly short when predictability for both spatial and temporal information is high. However, given that RT can include not only the amount of time needed to orient attention to the target, but also the amount of time to generate a response, we also recorded eye movements as a measure of overt attention. We examined whether temporal predictability could be used to orient overt attention more rapidly by measuring the time to fixate on the target after the target onset. In addition, if spatial predictability affects the orienting of attention, then high spatial predictability should allow attention to be oriented to the target location prior to its onset. If the target location is not known with certainty (i.e., there is not high spatial predictability) then attention cannot always be directed to the target before its onset.

### Method

#### Participants

Forty-five undergraduate students from Louisiana State University participated in this experiment in exchange for course credit. Demographic data was lost due to computer error for three participants. The remaining 42 participants had a mean age of 20 years and 12 were male.

#### Ethics Statement

This study was approved by the Institutional Review Board at the Louisiana State University, Baton Rouge. All participants gave written informed consent prior to participating in the experiment.

#### Apparatus

An SR EyeLink II eye tracker was used to present the stimuli, record key press responses, and collect eye movement data. The EyeLink recorded eye movements at a sampling rate of 250 Hz with a spatial resolution of approximately 0.5° using an infrared video-based tracking technology to compute the center and size of the pupils in both eyes. The head was stabilized by means of a chin rest located 47 cm from the monitor. During each trial, eye tracking began approximately 188 ms after a response was generated and continued until the next target appeared. This time lag is due to the inherent configuration of the Eyelink system. The eye tracker was re-calibrated prior to the commencement of each block to account for subtle changes in head position. The EyeLink system also recorded RT data for each trial.

#### Design and Procedure

The participants' task was to detect the appearance of a red square. They were instructed to pay close attention to the computer monitor and respond as quickly as possible when a target appeared on-screen. The target was a 20×20 pixel red square measuring 0.8 cm and subtending a 0.98° of visual angle at a viewing distance of 47 cm. The target was presented on a 19-inch CRT monitor set at a 1024×768 resolution. Participants were instructed to press either the left or the right trigger button on the SR Eyelink response controller (a Microsoft Sidewinder gamepad) as soon as the target appeared on the screen. ISI timing for the next target began immediately after the response. If the participant did not generate a response within 2000 ms of the onset of the target, the target was removed and the next ISI began. Participants were provided with the opportunity to rest between each block of trials.

Participants completed 9 blocks of 112 trials for a total of 1,008 trials. The level of spatial and temporal predictability was set for each block of trials. There were three levels of predictability for each variable (high, medium and low), yielding a 3 (temporal) ×3 (spatial) fully crossed, repeated-measures design (total of 9 different conditions). The order of the blocks was randomly determined for each participant.

For the different levels of spatial predictability, the number of possible target locations varied. There were one (high predictability), two (medium predictability), or four (low predictability) possible spatial locations. All targets appeared in one of the four quadrants of the screen. The four possible locations were the corners of a 14.5 cm×9.2 cm invisible square centered on the screen. From the center of the possible target locations, there was a 16.6° horizontal visual angle and a 10.2° vertical visual angle between target locations. Each location was 9.7° from the center of the screen. In the high predictability spatial condition, the target always appeared in one of the four possible locations. The location was chosen randomly for each participant and each block of high spatial predictability. In the medium predictability spatial condition, the target could appear in either of two diagonal quadrants. The set of diagonal locations was chosen randomly for each participant and each block of medium spatial predictability. In the low predictability spatial condition, the target could appear in any of the four quadrants. Within the low and medium levels of spatial predictability, the target appeared in the target locations with equal probability and the order of the spatial locations within a block was random.

For the temporal variable, the ISI between targets varied. Predictability was manipulated through the number of possible ISIs: one (high predictability: 1250 ms), two (medium predictability: 1000 ms, 1500 ms), or four (low predictability: 500 ms, 1000 ms, 1500 ms, 2000 ms). Within each level of temporal predictability, all possible ISIs occurred with equal probability, insuring that the average ISI for all trial blocks remained constant at 1250 ms. The order of ISIs across trials within a block was random.

### Results

#### The Effects of Spatial and Temporal Predictability on Reaction Time

RT was analyzed with a 3 (spatial predictability: high, medium, and low) ×3 (temporal predictability: high, medium, and low) repeated measures ANOVA. For the RT analysis, the following trials were excluded from analysis: A) the first trial of each block; B) trials for which a response was given within 99 ms after the target appeared; and C) trials for which no response was given within the 2000 ms time limit. Only 1.1% of all of the trials were excluded because a response was not given between 99 ms and 2000 ms, and a majority of these excluded trials (92.6%) were removed because the participant responded faster than 100 ms. The data were also analyzed using a logarithmic transformation (log_10_) of the RTs (including RTs lower than 99 ms). In addition, the data were examined using the low spatial/low temporal condition as a baseline for each participant and subtracting RTs in the other conditions from this baseline condition. Neither the log transform nor the baseline method produced results different from those using the raw RTs. For ease of interpretation and clarity, the results are presented using only the analysis of the raw RTs.

**Figure 1:**
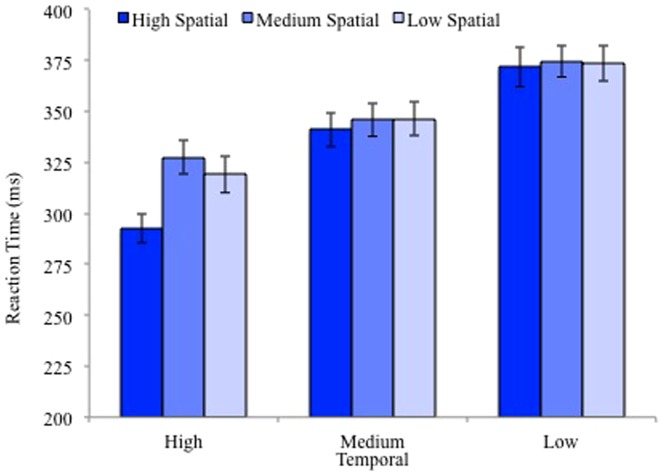
Reaction times from Experiment 1 for each level of temporal and spatial predictability. Error bars represent standard error of the mean.

Reaction time data is plotted in Figure 1. There was a main effect for temporal predictability (F(2,88)  = 140.35, MS = 122576.44, p<.001, η_p_^2^ = .76), a main effect of spatial predictability (F(2,88)  = 5.91, MS = 7656.32, p = .004, η_p_^2^ = .12), and a significant interaction between spatial and temporal predictability (F(2.95,129.98)  = 4.0, MS = 5358.84, p = .01, η_p_^2^ = .08, Greenhouse-Geisser corrected). Planned comparisons revealed an increase in RTs as temporal predictability decreased across all levels of spatial predictability (all p<.01). High spatial predictability RTs were significantly shorter than medium and low for the high temporal predictability condition (high vs. medium: t(44)  = 4.28, p<.001; high versus low: t(44)  = 3.8, p<.001). No other comparisons were significant (all p>.48).

**Figure 2:**
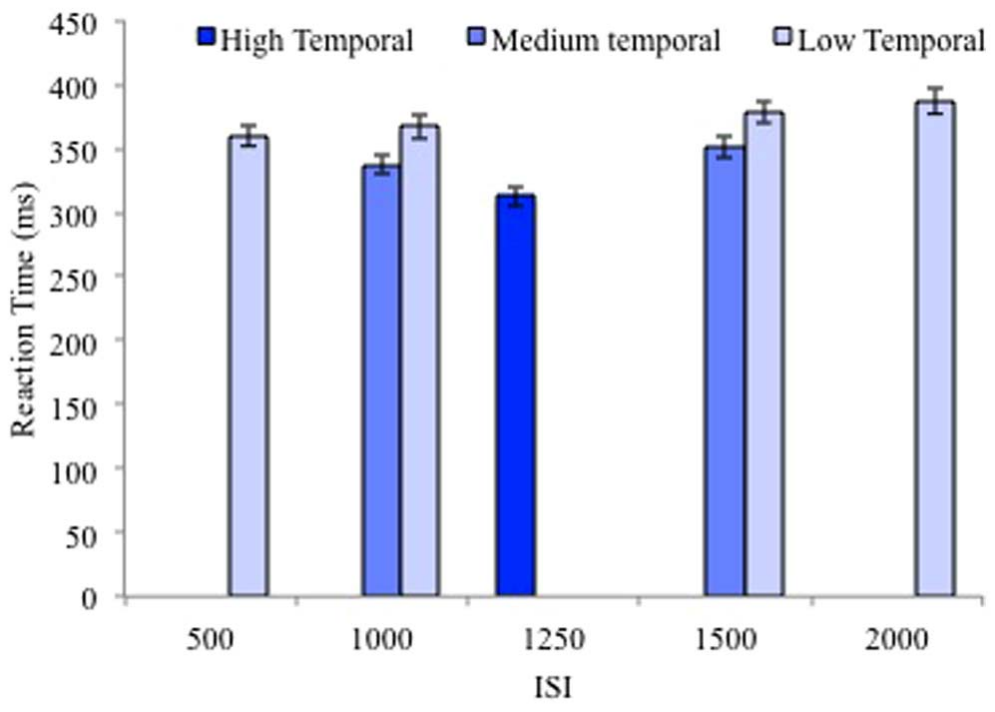
Average RT for each ISI at each level of temporal predictability in Experiment 1. RTs are averaged across the levels of spatial predictability within each level of temporal predictability. Error bars represent standard error of the mean.

RT data was also analyzed with ISI length as a factor (Figure 2) in order to examine the effect of temporal predictability within the context of the cumulative hazard function, which exists in simple-RT tasks without catch trials [13]. The cumulative hazard function predicts that RTs should decrease as ISI length increases; however, the temporal predictability hypothesis predicts that responses should be faster at the average ISI (1250, which occurred only in the high temporal predictability condition) rather than the longest ISI (2000 ms, which occurred only in the low predictability condition). A repeated measures ANOVA with ISI length as a within subjects factor with 5 levels (500, 1000, 1250, 1500, 2000) was conducted on RT averaged across all levels of spatial and temporal predictability (see Figure 2 for RT at ISIs at each ISI for level of temporal predictability, but averaged across levels of spatial predictability for visual simplicity). There was a main effect of ISI length (F(2.48,109.08)  = 141.94, MS = 53346.18, p<.001, η_p_^2^ = .76, Greenhouse-Geisser corrected). The linear and quadratic effects of ISI length were significant (ps<.001) due to RTs decreasing as ISI length increased from 500 ms to 1250 ms and by RTs increasing as ISI increased from 1250 ms to 2000 ms (see Figure 2). Therefore, RTs for the average ISI (1250 ms), used only in the high temporal predictability condition, were shortest. These results suggest that cumulative hazard functions for the low and medium temporal predictability conditions did not lead to the differences in RTs across the temporal predictability conditions. Effectively, the shortest ISIs in the low and medium temporal predictability conditions were not driving the temporal predictability effect; RTs for all ISIs in the low and medium temporal predictability conditions (500 ms, 1000 ms, 1500 ms, and 2000 ms) were longer than the RTs for the 1250 ms ISI used in the high temporal predictability condition.

#### The Effects of Spatial and Temporal Predictability on Visual Fixations

A fixation was classified when eye movement velocity was below the threshold of 30°/s and the length of time for which the eye remained below this threshold was the fixation duration. Fixations were coded as on a target location if the fixation fell within a 120×120 pixel square centered on the target.

It is possible that participants adopted a strategy of simply leaving their eyes on the previous target location during the ISI regardless of spatial predictability. If this were the case, this strategy would lead to faster RTs when spatial predictability was high because the target was more likely to appear in the previous target location. If participants used this strategy, it would be expected that the dwell time (sum of all fixation durations) would be greater on the previous target location than other target locations or non-target locations, regardless of spatial predictability. In contrast, it is possible participants used the learned probability information to allocate overt attention to the probable target location and therefore only used this strategy of leaving the eyes on the previous target location when spatial predictability was high. If this were the case, it would be expected that the dwell time would be greater on previous target locations only when spatial predictability was high.

Therefore, the average dwell times of fixations were calculated on: 1) the previous target location, 2) another target location (all three remaining possible target locations were included regardless of whether targets actually appeared in the locations for a given block of trials), and 3) non-target locations were calculated (see Table 1). An ANOVA on the dwell times on the previous target location was performed. There was a main effect of spatial predictability (F(1.25,86.52)  = 94.24, MS = 18288324.52, p<.001, η_p_^2^ = .68, Greenhouse-Geisser corrected) reflecting increased dwell time as spatial probability increased. Both linear and quadratic trends for spatial predictability were significant (ps<.001) due to a larger difference between high and medium spatial predictability and smaller differences between medium and low spatial predictability. There was no main effect for temporal predictability (F(2,88)  = .49, MS = 30808.42, p = .62, η_p_^2^ = .01). The interaction was not significant (F(2.36, 103.62)  = .65, MS = 77721.09, p = .65, η_p_^2^ = .63, Greenhouse-Geisser corrected).

**Table 1:** Average dwell time on the previous target location, one of the other three possible target locations, and a non-target location during the ISI for Experiment 1.

	Previous Target	Another Target	No Target
**HS/HT**	743.78 (73.26)	0.26 (.21)	559.15 (70.53)
**HS/MT**	705.65 (78.86)	0.27 (.14)	639.27 (75.14)
**HS/LT**	789.34 (71.6)	2.05 (1.67)	561.61 (72.11)
**MS/HT**	300.55 (49.02)	5.76 (3.06)	882.66 (52.02)
**MS/MT**	285.32 (40.08)	2.87 (1.94)	908.33 (47.8)
**MS/LT**	261.72 (39.03)	7.31 (1.7)	932.18 (46.24)
**LS/HT**	236.09 (37.71)	3.92 (0.85)	933.49 (42.71)
**LS/MT**	199.15 (24.54)	3.99 (1.09)	992.34 (36.56)
**LS/LT**	190.87 (24.94)	5.11 (1.39)	999.01 (35.47)

*Note*. Times are in ms and standard error of the mean is in parentheses. H =  high, M =  medium, L =  low, S =  spatial, T =  temporal.

**Figure 3:**
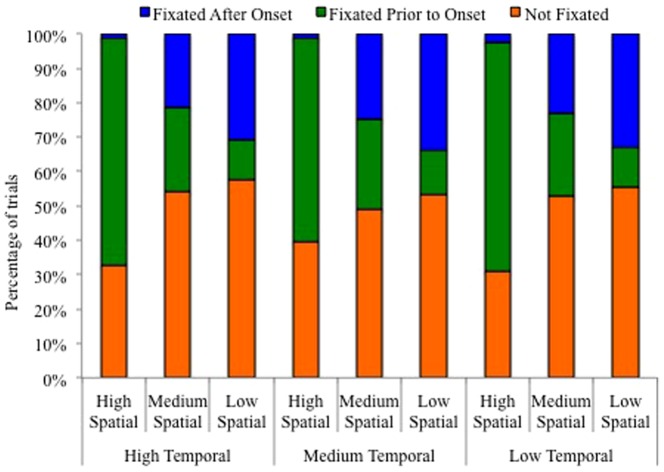
The percentage of trials when the target location was fixated prior to target onset, fixated after target onset, and not fixated at all for all levels of temporal and spatial predictability.

In order to further examine the effects of spatial and temporal predictability on the orienting of attention, an examination was performed of the point in the trial when the target was fixated, if it was fixated at all. There were three possibilities for each trial: 1) the target was not fixated, suggesting covert attention was used to detect the target, 2) the target was fixated prior to and during the onset of the target, or 3) the target was fixated after the target onset. The proportion of each possibility was calculated for each condition (see Figure 3). Based on the dwell time analysis that revealed the greatest overall dwell time in the high spatial predictability condition and the data in Figure 3 that shows a greater proportion of trials where the target was fixated prior to the target onset when there was high spatial predictability, it appears that high spatial predictability allowed participants to orient attention to the location of the target before the target even appeared.

However, high temporal predictability facilitates faster overt orienting of attention to the location of the target after the onset of the target. To examine this hypothesis, the amount of time between when the target appeared and when fixation arrived at the target location was examined. As a result, only trials in which fixation on the target occurred after target onsets were included (see Figure 3). The high spatial condition was excluded from the analysis because the target was rarely fixated after target onset on these trials. Therefore, a 2 (medium spatial, low spatial) ×3 (high temporal, medium temporal, low temporal) ANOVA was conducted. Participants with missing data in any condition (i.e., there were no trials in which the target was fixated after onset) were excluded, leaving data from 33 participants for this analysis. There was a main effect for temporal predictability (F(2,64)  = 7.75, MS = 61.59, p = .001, η_p_^2^ = .195), but no main effect of spatial predictability (F(1,32)  = 3.7, MS = 2173.87, p = .063, η_p_^2^ = .104), and no interaction between spatial and temporal predictability (F(1.7,63.9)  = .19, MS = 196.8, p = .83, η_p_^2^ = .006, Greenhouse-Geisser corrected). The linear trend for temporal predictability was significant (p = .002) due to the decrease in the amount of time to fixate the target after target onset as temporal predictability increased. This shows that temporal predictability actually allows participants to shift overt attention to the target more quickly. Because the target location is unknown in the medium and high spatial predictability conditions, the eye movements cannot be oriented to the target location until after its onset. Therefore, temporal predictability can be used to orient attention to the target location and is therefore important for orienting attention.

#### Is Strategic Allocation of Overt Attention Responsible for the RT Effects?

In order to determine if the ability to use spatial predictability to orient attention to the target location prior to target onset was important for the effect of spatial predictability on RT, an examination of RTs for trials when participants were not already looking at the target location at the time of target onset was conducted. Effectively, only trials for which the target was fixated after target onset or not at all were included. Some participants had blocks of trials in which they were always looking at the target location at the onset of the target. These three participants were excluded from this analysis. There was a main effect for temporal predictability (F(2,82)  = 111.15, MS = 134921.13, p<.001, η_p_^2^ = .73), where RTs increased as predictability decreased. There was no main effect of spatial predictability (F(2,82)  = .1, MS = 217.37, p = .9, η_p_^2^ = .003). The interaction was not significant (F(2.79, 114.48)  = 2.43, MS = 5547.83, p = .07, η_p_^2^ = .06, Greenhouse-Geisser corrected).

### Discussion

Temporal and spatial predictability led to faster responses to the target, supporting the hypothesis that participants were capable of determining target predictability based on the inherent pattern of presentation. Furthermore, the effect of temporal predictability (η_p_^2^ = .76) was larger than the effect of spatial predictability (η_p_^2^ = .12). While temporal predictability facilitated performance across all levels of spatial predictability, the effect of spatial predictability was primarily evident only when both spatial and temporal predictability were high. This suggests that the effect of spatial predictability is mediated by temporal predictability. Effectively, the advantage of knowing “where” a stimulus will appear is contingent on knowing “when” it will appear.

In order to more closely examine the effects of spatial and temporal predictability on the allocation of attention, fixation dwell time during the ISI and the amount of time between target onset and when fixations arrived at the target was examined. Spatial predictability allowed participants to visually fixate on the target location prior to its onset. Temporal predictability, on the other hand, allowed fixations to arrive on the target more quickly after its onset. These results demonstrate that spatial predictability is learned and used to strategically allocate and maintain attention on probable target locations. Furthermore, temporal predictability is also learned and used to compensate for increased uncertainty in target location by allowing for more rapid shifts in visual fixation after the target has appeared. Although high spatial predictability led to fixations being oriented at the target location regardless of the level of temporal predictability, RTs were faster for high spatial predictability only when temporal predictability was also high. This suggests that without prior anticipation of when a target will appear, the allocation of overt attention to the correct target location is not sufficient to improve RTs.

One possible explanation for this interaction between spatial and temporal predictability is that covert attention may have wandered from the task or from the fixation location at the time of target onset. The fixation dwell time suggests that participants were likely to maintain their fixation at the target location throughout the entire block of high spatial probability trials. Yet, even though fixation was at the target location, attention was not necessarily allocated at the fixation location or the task at the time of target onset. Consistent with previous literature demonstrating temporal orienting affects with symbolic endogenous cues [1], the current findings suggest an underlying dynamic process of attention orientation where the timing of attention allocation is essential to the speed of a response.

Although there is also evidence of an effect of temporal predictability on overt orienting of spatial attention, it is possible that when there is only a single possible target location, the participants exploited temporal predictability by simply making their response patterns more rhythmic (i.e., pressing the response button at regular intervals), reducing the need to attend to the target and allowing for faster RTs. In Experiment 2, a discrimination task was used in which the target must be identified before a response is given, making response preparation more difficult.

## Experiment 2

The target discrimination task used in Experiment 2 varied from the detection task in two important ways. First it was not possible to pre-plan a response prior to target onset. This removes the possibility of response preparation contributing to the temporal predictability effect. Second, the cognitive load of the task is higher because identification of the target and a decision about which response to give must be made. The ability to incidentally learn and use spatial and temporal probability information may depend on the cognitive resources available. Learning probability information requires attention [20] [25] [26] and therefore, when cognitive load is high for the task, there may be less of an influence of temporal and/or spatial information and less of a relationship between the two. Based on previous research, the use of temporal cues will be more difficult when the cognitive load of the task is high. Several studies have indicated that temporal orienting (i.e., strategically orienting attention to the target location based on knowledge of when the target is likely to appear) is less likely to occur when cognitive resources are occupied [34] [13].

Correa et al. [13] found that the effect of temporal predictability on RTs was not present in a discrimination task when interpretation of a temporal cue required complex stimulus-response (S-R) pairings for the temporal cue (green cue indicates a short SOA and a red cue indicates a long SOA), but was present in a discrimination task requiring simpler S-R pairings (short line indicates a short SOA and a long line indicates a long SOA). Therefore, the ability to use temporal predictability to influence the spatial cueing affect appears to require cognitive resources. This suggests that when the cognitive load of the task is higher (e.g., in a discrimination task as compared to a detection task), the effect of temporal predictability may be lower.

The design for Experiment 2 was the same design as in Experiment 1, with the only addition being that participants have to determine if the target is red or green and press a different key for each color. This manipulation tests whether this additional layer to stimulus-response pairing alters the effects of spatial and temporal predictability on orienting attention.

### Method

#### Participants

Forty-one undergraduate students from Louisiana State University participated in this experiment in exchange for course credit. Demographic data was lost due to computer error for one of the participants. The remaining 40 participants had a mean age of 20.5 years and 9 were male.

#### Ethics Statement

This study was approved by the Institutional Review Board at the Louisiana State University, Baton Rouge. All participants gave written informed consent prior to participating in the experiment.

#### Design and Procedure

This experiment was the same as Experiment 1 except that participants performed a target discrimination task instead of a target detection task. The target could be either red or green and the participant pressed one button for a red target and another button for a green target. Half of the trials in each block contained a red target and half contained a green target.

### Results

Data analysis was conducted similarly to Experiment 1. Trials were incorrect (3.14% of the trials) if a response was not given between 99 ms and 2000 ms from the onset of the target or if the wrong button was pressed. Almost all of the excluded trials were trials in which the participant pressed the wrong key (3.10% of all the trials. Percent of trials excluded for each level of temporal/spatial predictability: high/high  = 2.72%, high/medium  = 4.31%, high/low  = 3.73%, medium/high  = 2.55%, medium/medium  = 3.37%, medium/low  = 3.24%, low/high  = 2.25%, low/medium  = 2.83%, low/low  = 2.9%). The results were also analyzed using a log-transform (including RTs lower than 99 ms). In addition, the results were conducted by using the low spatial/low temporal condition as a baseline for each participant and subtracting RTs in the other conditions from this baseline condition. Neither the log transform nor the baseline method produced results different from using the raw RTs, so for ease of interpretation, the raw RTs are used for the analyses below.

#### The Effects of Spatial and Temporal Predictability on Reaction Time

**Figure 4:**
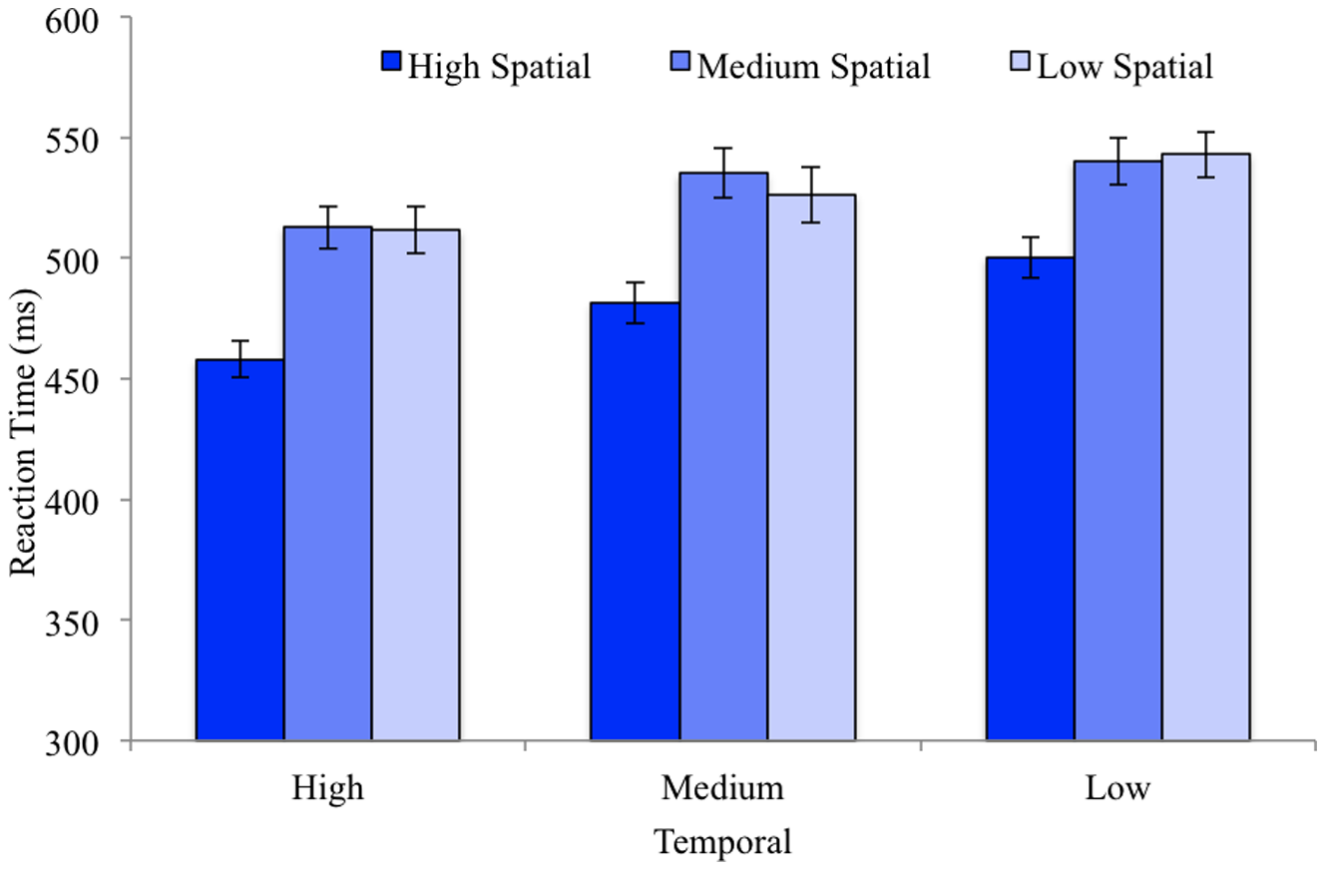
Reaction times from Experiment 2 for each level of temporal and spatial predictability. Error bars represent standard error of the mean.

There was a main effect for temporal predictability (F(2,80)  = 23.99, MS = 35146.49, p<.001, η_p_^2^ = .38), a main effect of spatial predictability (F(2,80)  = 73.03, MS = 95483.84, p<.001, η_p_^2^ = .65), but no interaction between spatial and temporal predictability (F(4,160)  = 1.25, MS = 943.77, p = .29, η_p_^2^ = .03). The linear effect of temporal predictability was significant (p<.001) due to RTs increasing as temporal predictability decreased (see Figure 4). The linear and quadratic effects were significant for spatial predictability (ps<.001) due to reaction times increasing primarily from high to medium spatial predictability, but not from medium to low spatial predictability.

**Figure 5:**
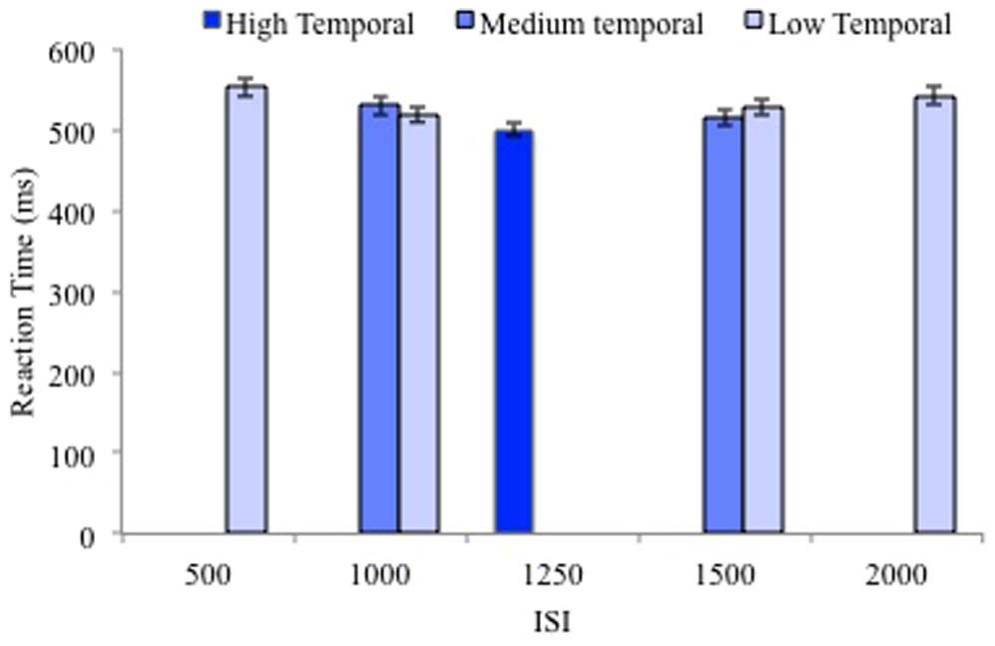
Average RT for each ISI at each level of temporal predictability in Experiment 2. RTs are averaged across the levels of spatial predictability within each level of temporal predictability. Error bars represent standard error of the mean.

RT data were also analyzed with a repeated measures ANOVA with ISI length as a within subjects factor with 5 levels (500, 1000, 1250, 1500, 2000). There was a main effect of ISI length, F(2.51,100.48)  = 30.16, MS = 26650.05, p<.001, η_p_^2^ = .43, Greenhouse-Geisser corrected. The linear effect was not significant (p = .11), but the quadratic effect was significant (p<.001). This main effect was driven in part by RTs decreasing as the ISI length increased from 500 ms to 1250 ms and by RTs increasing from 1250 ms to 2000 ms ISIs (see Figure 5). This is the pattern expected given that predictability is highest for the 1250 ms ISI (used only in the high predictability condition), moderate for the 1000 ms and 1500 ms ISIs (used in the medium and low predictability conditions), and lowest for the 500 ms and 2000 ms ISIs (used only in the low predictability condition). There was no significant difference between the 500 ms and 2000 ms ISIs (p = .1) suggesting, as suspected, that there was no effect of a hazard function in the discrimination RTs in the low temporal predictability condition. Most importantly, RTs in the 1250 ms ISI trials were faster than the RTs in the other ISI trials (all Ps<.001), demonstrating that high temporal predictability led to faster RTs than the RTs in all of the ISIs used in the low and medium temporal predictability conditions.

#### The Effects of Spatial and Temporal Predictability on Visual Fixations

The average dwell times of fixations on the previous target location, another target location (all three remaining possible target locations were included regardless of whether targets actually appeared in the locations for a given block of trials), and non-target locations are presented in Table 2. An ANOVA was conducted for the dwell times on the previous target location. There was a main effect of spatial predictability (F(1.47, 61.54)  = 65.61, MS = 12686012.46, p<.001, η_p_^2^ = .62, Greenhouse-Geisser corrected), with longer dwell times on the previous target location with increasing spatial predictability. The quadratic trend was significant (p<.001) due to longer dwell times for high spatial predictability than for medium spatial predictability, but no difference between the medium and low spatial predictability conditions. Therefore, participants were more likely to leave fixations on the previous target location in the high spatial predictability condition than in the medium or low spatial predictability conditions. There was no main effect for temporal predictability (F(1.47, 61.54)  = .61, MS = 74623.87, p = .5, η_p_^2^ = .02, Greenhouse-Geisser corrected) and no interaction (F(2.66, 106.18)  = .63, MS = 71610, p = .65, η_p_^2^ = .58, Greenhouse-Geisser corrected).

**Table 2:** Average dwell time on the previous target location, one of the other three possible target locations, and a non-target location during the ISI for Experiment 2.

	Previous Target	Another Target	No Target
**HS/HT**	839.13 (83.9)	0.17 (0.14)	564.52 (80.75)
**HS/MT**	750.85 (82.12)	0.25 (0.14)	665.61 (79.44)
**HS/LT**	740.63 (88.21)	0.12 (0.09)	679.39 (85.24)
**MS/HT**	361.05 (51.89)	3.26 (1.59)	897.37 (59.01)
**MS/MT**	346.10 (50.06)	4.04 (1.8)	911.20 (60.81)
**MS/LT**	373.04 (50.45)	5.95 (2.16)	853.76 (59.59)
**LS/HT**	267.68 (37.06)	4.91 (2.42)	957.23 (48.07)
**LS/MT**	276.31 (39.58)	3.28 (1.3)	966.02 (52.80)
**LS/LT**	230.14 (34.43)	6.76 (4.21)	992.94 (45.62)

*Note*. Times are in ms and standard error of the mean is in parentheses. H =  high, M =  medium, L =  low, S =  spatial, T =  temporal.

**Figure 6:**
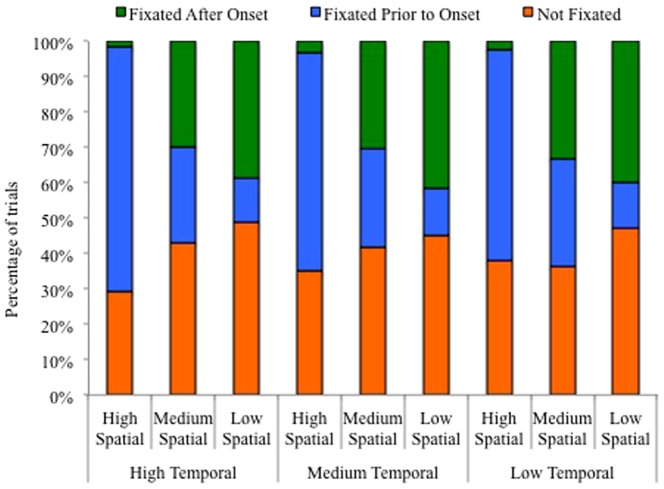
The percentage of trials when the target location was fixated prior to target onset, fixated after target onset, and not fixated at all for all levels of temporal and spatial predictability.

As in Experiment 1, fixations during target presentation were examined (see Figure 6). To examine the effect of temporal predictability on attention allocation, the amount of time from target onset until fixation on the target was calculated for the trials in which the target was fixated after target onset. The high spatial condition was excluded from the analysis because the target was rarely fixated after onset on these trials. Therefore, a 2 (medium spatial, low spatial) ×3 (high temporal, medium temporal, low temporal) ANOVA was conducted. Participants with missing data in any condition (i.e., there were no trials for that condition in which the target was fixated after onset) were excluded, leaving data from 33 participants for this analysis. There were no main effects for temporal predictability (F(2,64)  = .86, MS = 616.95, p = .43, η_p_^2^ = .026), or spatial predictability (F(1,32)  = 1.57, MS = 13.02.63, p = .219, η_p_^2^ = .047), and no interaction between spatial and temporal predictability (F(2,64)  = .79, MS = 582.72, p = .46, η_p_^2^ = .024).

#### Is Strategic Allocation of Overt Attention Responsible for the RT Effects?

An examination of RTs on the trials when overt attention was not on the target at the time of target onset was performed. Therefore, only trials for which the target was fixated after target onset or the target was never fixated were included. Some participants had blocks of trials in which all trials had fixations on the target location at the time of target onset target. These five participants were excluded from this analysis. There was a main effect for temporal predictability (F(2,70)  = 16.87, MS = 33975.13, p<.001, η_p_^2^ = .33), and a main effect of spatial predictability (F(1.66,58.04)  = 28.6, MS = 60643.87, p<.001, η_p_^2^ = .45, Greenhouse-Geisser corrected). The interaction was not significant (F(3.21, 112.42)  = 1.19, MS = 1751.14, p = .32, η_p_^2^ = .03, Greenhouse-Geisser corrected). The linear trend for the temporal effect was significant (p<.001) due to RTs increasing as temporal predictability decreased, and the linear and quadratic trends were significant (ps<.001) for the spatial effect due to a larger increase in RTs from high to medium spatial predictability than from medium to high spatial predictability.

### Discussion

As in the detection task used in Experiment 1, predictability contained in the inter-trial relationships of when and where a target is likely to occur led to shorter RTs. However, unlike in Experiment 1, the effect of spatial predictability (η_p_^2^ = .65) was higher than the effect of temporal predictability (η_p_^2^ = .38), and there was no interaction between temporal and spatial predictability on RTs. As in Experiment 1, temporal predictability facilitated performance across all levels of spatial predictability, but the effect was much smaller than that in Experiment 1 (η_p_^2^ = .76). Contrary to the effect of spatial predictability in Experiment 1, that was evident only when both spatial and temporal predictability were both high, in Experiment 2 high spatial predictability speeded RTs across all levels of temporal predictability. Therefore, in a sustained attention task requiring target discrimination, the effects of spatial and temporal predictability are independent of each other.

Similar to Experiment 1, the effect of temporal predictability on RTs outweighed the effects of the cumulative hazard function of RT and ISI length [28] [29]. RTs in the 1250 ms trials were faster than RTs at all other ISI lengths, demonstrating that slower RTs on the shorter ISI trials in the medium and low predictability conditions were not the sole cause to the temporal predictability effect. The speeded RTs during the high temporal predictability condition also cannot be explained by faster RTs for shorter ISIs [35]. RTs during the 500 and 1000 ms ISI trials were significantly slower than RTs during the 1250 ms ISI trials. These results demonstrate that the effect of temporal predictability is evident even after the possible effects of ISI duration are accounted for.

The discrimination task prevented anticipatory response preparation because the response was not known until the stimulus was identified. As such, rhythmic responses are not likely to have contributed to faster RTs when temporal predictability was high. Consistent with this, the effect of temporal predictability was smaller in this experiment than in Experiment 1. In addition, temporal predictability did not improve the ability to orient attention to the target after onset when spatial predictability was low. These results suggest that the complexity of the discrimination task prevented the use of temporal predictability for allocation of attention to the target location more quickly when the target location was not certain. Furthermore, the effects of spatial and temporal RT remained significant even when the trials in which attention was allocated to the target location prior to target onset were excluded. This suggests that the effects of spatial and temporal predictability on performance in a discrimination task are not solely the result of a strategic allocation of overt attention.

## General Discussion

Across both discrimination and detection tasks, the findings were consistent with the previous research showing that RT decreased under greater levels of target predictability. Even without the presence of an overt exogenous or endogenous pre-target cue, RTs decreased with increasing target predictability. This supports the hypothesis that when the amount of information that must be processed prior to execution of the response is increased, the response itself is slowed [4] [5]. Although both high spatial and temporal predictability reduced RTs, their effects and interaction were task-dependent. The results also supported the hypothesis that stimulus predictability can alter the orientation of attention, as evidenced by the changes in visual fixation patterns.

A unique aspect of the current study is that it demonstrates that the predictability of when and where a target is likely to appear can be learned through inter-trial relationships and used to reduce RT. In contrast, most research examining similar questions about spatial and temporal predictability utilized cues to present the predictability information [13] [1] [10] [14] [7] [16] [11] [8] [9]. The finding that predictability can be obtained through experience is consistent with Doherty et al. [2], who demonstrated that previous experience with a target could be used to predict when and where a target will appear from behind an occluding object. The sustained attention task used in this study demonstrated that accumulated probability information from repeated location and onset times of previous targets could be used by participants to incidentally learn both the temporal and spatial predictability of a target. Most importantly, the current study demonstrates that this acquired spatial and temporal information influences performance differently for detection and discrimination sustained attention tasks.

Although spatial and temporal predictability led to faster RTs across both tasks, their effects and interaction differed. For the detection task, the effect size of temporal predictability was largest, alongside a significant interaction between spatial and temporal predictability. However, when participants performed the discrimination task, the effects of target predictability in space and time where independent and the effect of spatial predictability was larger than the effect of temporal predictability. The visual fixation analyses suggested that these differences were due to an interaction between spatial and temporal predictability on attention orientation in the detection task, but no such interaction in the discrimination task. In both tasks, high spatial predictability allowed fixations to be oriented to the target location during the ISI and therefore, prior to target onset. However, in the detection task, this lead to faster RTs only when temporal predictability was high, while in the discrimination task, spatial predictability sped RTs even when overt attention was not directed to the target prior to target onset. Furthermore, high temporal predictability led to faster orienting of overt attention to the target only in the detection task. This demonstrates that information useful for advanced orienting of attention in both space and time are needed in order to gain an interactive benefit for spatial and temporal predictability on RT.

A significant effect of spatial predictability was found in both tasks. Specifically, RTs were faster when spatial predictability was high. Analysis of eye movements demonstrated that this was a result of the strategic allocation of attention based on the learned probability information rather than an artifact of sustained attention on the previous target location regardless of the spatial probability. Specifically, if participants simply left fixation on the previous target location for all blocks of trials, this would lead to more fixations on the target prior to target onset regardless of the level of spatial predictability, which could speed RTs in the high spatial predictability condition. If this were the case, we would expect the average dwell time on the previous target location to be higher than dwell times on other possible target locations across all levels of spatial predictability. However, in both experiments, dwell time on the previous target location was highest in the high spatial probability condition demonstrating that this was a strategic allocation of overt attention based on the learned probability information.

A significant effect of temporal predictability was also found in both tasks. Specifically, RTs were faster when temporal predictability was high. The temporal predictability effect in Experiment 1 could have been impacted by response preparation because in detection tasks, the correct response is known prior to the onset of the target. The temporal predictability effect in Experiment 1 was also impacted by the allocation of overt attention to the target location. Specifically, when temporal predictability was high, participants were able to direct fixations to the target location more quickly than when temporal predictability was low. In the discrimination task of Experiment 2, response preparation was not possible because the target needed to be identified prior to the generation of a response, and fixations were not allocated to the target more readily after target onset when temporal predictability was high.

Why did the learned temporal probability information fail to lead to strategic allocation of overt attention in the discrimination task? The results from Correa et al. [13] suggest that the added cognitive load of the discrimination task and/or the probability information may prevent the use of the temporal information for orienting attention. Correa et al. [13] found that when the temporal cue for the onset time of the target (an X or an O presented at fixation with no spatial variability) required a more complex S-R pairing, there was no effect of temporal predictability. It is possible that maintaining the response parings for red vs. green targets in memory made the benefits of temporal information more difficult to encode or use. It is also possible that the increased complexity of the predictability information had an effect. Because a continuous performance task was employed, the discrimination task has an added layer to the probabilities of event occurrences. In the detection task, there is only the possibility that a target appears in location X at time Y. During the discrimination task, there is the possibility that a target of color Z will appear in location X at time Y. For example, when there are two ISIs and two spatial locations, the joint probability of the target being on the left and after a 1000 ms is 0.25. Adding the third layer of color, the probability of left +1000 ms+ green would be 0.125, altering the joint probability of occurrence. Per the original concept of information processing in cognition [3] [4], the addition of the discrimination task and/or the increased complexity of the probability information would lead to an increased cognitive load, as there is now a greater level of uncertainty that has to be overcome. While the ability to accumulate simple probability distributions through inter-trial relationships is now documented, the extent of the human ability to acquire and capitalize on complex joint and conditional probabilities purely from inter-trial patterns of repeated exposures to visual stimuli during a sustained attention task is not yet known and is a question for future research.

If there was no temporal advantage to attention orientation in Experiment 2, what caused the main effect of temporal predictability on RTs? It is possible that the effect of temporal predictability in Experiment 2 was driven by an increase in proactive inhibitory control in this experiment as compared to Experiment 1 [36] [37]. Another possibility is that temporal predictability affected perceptual processes involved with the identification of the targets [38]. It is also possible that temporal predictability cues increased the overall alertness of the participant [39], but not the attentional preparation that may be necessary for actions related to the stimulus, like generating an eye movement [1]. Temporal predictability can also lead to more automatic effects on attention rather than controlled effects as demonstrated by the sequential effects found when consecutive trials have the same ISI. Sequential effects (the effects of trial n on trial n+1) are most likely in the high predictability condition because consecutive trials with the same ISI are ubiquitous in the high temporal predictability condition and are more likely in the medium temporal than the low temporal predictability conditions. Sequential effects appear to be dissociable from orienting effects and are more automatic and less strategic [13] [38]. Most importantly, regardless of which of these is causing the main effect of temporal predictability on RTs, the effect is independent of spatial predictability in the discrimination task.

Overall, our results provide initial evidence that spatial and temporal predictability can be accumulated through inter-trial relationships, and: A) can speed up response times and B) can alter overt attention allocation. There are limitations and open questions requiring future research. First is a question of whether the sequence of events, rather than the accumulated probability distribution, is the source of information used to determine predictability? This cannot be addressed within the current paradigm as the target occurrences were randomized and were different from one participant to the next. Furthermore, as mentioned earlier, there is a potential confound of sequential effects of adjacent trials as higher probability of occurrence would mean many more adjacent trials with similar locations and durations, preventing a clear separation of occurrence from sequences at this time. Considering both aspects, it does raise an important question as to whether the lack of ability to distinguish predictable from random sequences (see [40]) might arise from the fact that accumulated probability of event occurrences takes precedence over sequences in the process of orienting attention to external stimuli. Second is a question of how discrimination tasks affect predictability. Unlike the detection task, the randomization of red vs. green targets alters the sequence and the joint and conditional probabilities. Exploring this phenomenon more carefully would require systematic manipulation of the co-occurrence of different location and ISI with that of the discrimination task. It is still unclear as to how adding a greater number of even incongruous stimulus-response pairs would affect stimulus predictability overall.
